# Combining BTK inhibitors with BCL2 inhibitors for treating chronic lymphocytic leukemia and mantle cell lymphoma

**DOI:** 10.1186/s40364-022-00357-5

**Published:** 2022-04-04

**Authors:** Jing Zhang, Xueying Lu, Jianyong Li, Yi Miao

**Affiliations:** 1 Department of Hematology, the First Affiliated Hospital of Nanjing Medical University, Jiangsu Province Hospital, Nanjing, China; 2 Key Laboratory of Hematology of Nanjing Medical University, Nanjing, China; 3 Pukou CLL Center, Nanjing, China; 4 National Clinical Research Center for Hematologic Diseases, the First Affiliated Hospital of Soochow University, Suzhou, China

**Keywords:** BTK inhibitors, BCL2 inhibitors, Chronic lymphocytic leukemia, Mantle cell lymphoma

## Abstract

The advent of BTK inhibitors has changed the treatment of patients with chronic lymphocytic leukemia (CLL) and mantle cell lymphoma (MCL). The first-in-class BTK inhibitor ibrutinib has shown remarkable therapeutic effects and manageable toxicities in multiple clinical trials. The second-generation BTK inhibitors, including acalabrutinib and zanubrutinib, also show remarkable efficacies. However, using BTK inhibitors as monotherapies requires continuous treatment. Resistance to BTK inhibitors and severe side effects unavoidably occur during BTK inhibitor monotherapy, frequently resulting in treatment failure. The addition of the BCL2 inhibitor venetoclax to BTK inhibitor may improve the therapeutic effects and result in deeper responses, providing a potential fixed-duration treatment, especially for patients with CLL. In this review, by focusing on CLL and MCL, we discussed the rationale for the combinational use and summarized the current data on the combinations of BTK inhibitors and venetoclax in patients with CLL and MCL.

## Background

The advent of BTK inhibitors has revolutionized the treatments of B cell malignancies, especially chronic lymphocytic leukemia/small lymphocytic lymphoma (CLL/SLL) and mantle cell lymphoma (MCL). The first-in-class BTK inhibitor, ibrutinib, was firstly approved for the treatment of patients with relapsed/refractory (R/R) MCL. This approval was based on a study of previously heavily treated patients with MCL, in which ibrutinib monotherapy induced responses in 68% of patients, including 21% of patients achieving complete remission (CR) [1]. The therapeutic efficacy of BTK inhibitors is even more impressive in patients with CLL/SLL. In the phase 3 RESONATE trial, ibrutinib significantly improved the progression-free survival (PFS) and overall survival (OS) of patients with R/R CLL/SLL, including those with TP53 aberrations [2]. Another four randomized phase 3 trials demonstrated that ibrutinib remarkably prolonged PFS of treatment-naïve (TN) patients with CLL/SLL as compared to chemoimmunotherapy [3–6]. These large randomized phase 3 trials established ibrutinib as the standard treatment of patients with CLL/SLL, in both R/R and first-line settings. The second-generation BTK inhibitors, including acalabrutinib and zanubrutinib, have also similar therapeutic effects in patients with CLL/SLL or MCL [7–9].

**Fig. 1:**
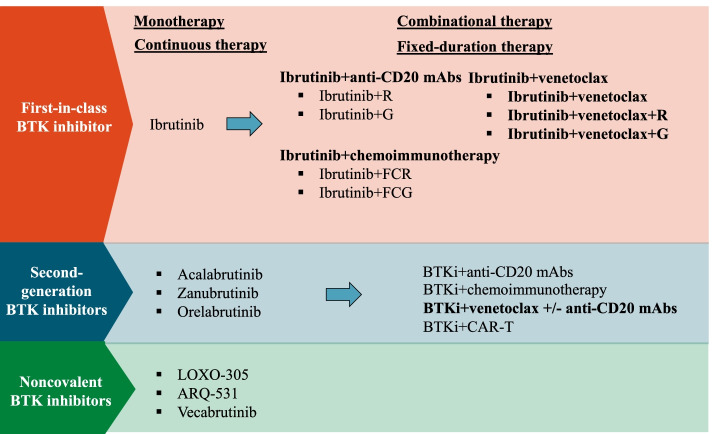
Ibrutinib is the first-in-class BTK inhibitor, followed by second-generation and noncovalent BTK inhibitors. BCL2 inhibitor venetoclax, anti-CD20 mAbs, chemoimmunotherapy, and CAR-T therapy have been combined with BTK inhibitors for fixed-duration treatment. BTKi, BTK inhibitors; CAR-T, chimeric antigen receptor T-cell; FCR, fludarabine, cyclophosphamide and rituximab; FCG, fludarabine, cyclophosphamide and obinutuzumab; G, obinutuzumab; mAbs, monoclonal antibodies; R, rituximab

Despite of the effectiveness of BTK inhibitors in patients with MCL or CLL/SLL, some challenges exist while using BTK inhibitors as monotherapy. Nearly one-third of patients with R/R MCL did not respond to ibrutinib monotherapy. And even for those responders, the median duration of response was less than 2 years [10], suggesting that ibrutinib monotherapy may be inadequate to provide long-term disease control of R/R MCL. Although the response rates are very high in CLL patients treated with ibrutinib monotherapy, most responses are partial remission (PR) and CR is infrequent. And there is a continued risk of disease relapse even with continuous use of ibrutinib, especially in those patients with high-risk features [11, 12]. Additionally, adverse events related to ibrutinib, most of which are caused by off-target inhibiting effect, could occur in CLL/SLL patients who receive ibrutinib treatment. Severe adverse events may lead to discontinuation of ibrutinib use, which is always associated with poor prognosis [13]. Although the second-generation BTK inhibitors are more selective than ibrutinib, side effects are inevitable while using these agents. Furthermore, the high cost caused by the continuous use of BTK inhibitors may also pose a challenge in clinical practice. The evolution of BTK inhibitors and BTK inhibitors-based combinational therapy is summarized in Fig. 1.

BCL2 is an anti-apoptotic protein that renders cells resistant to apoptosis. The BCL2 dysregulation is a key process in the pathogenesis of B cell lymphoma [14]. The oral BCL2 antagonist, venetoclax, is highly effective in patients with CLL/SLL [15, 16]. It has also demonstrated significant efficacy in MCL [17, 18]. The addition of venetoclax to ibrutinib increases the depth of response and may induce a longer duration of remission in patients with CLL and MCL. For patients with CLL/SLL, this combination strategy also provides a fixed-duration therapeutic option. In this review, we discussed the rationales for combining BTK inhibitors and BCL2 inhibitors for treating CLL/SLL and MCL. And we summarized the current data from the relevant clinical trials and discussed the future directions of this combination strategy.

### Mechanisms of synergy

#### CLL/SLL

**Fig. 2:**
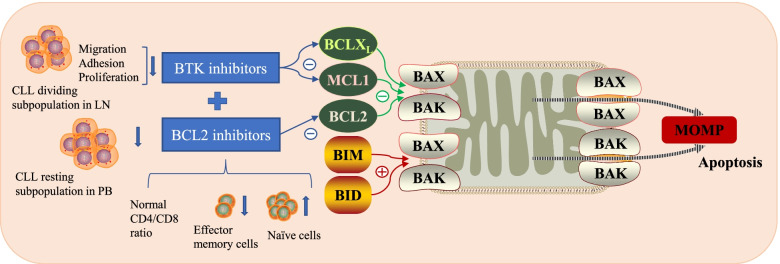
The distinct and complementary mechanisms of ibrutinib and venetoclax make the combination rational in CLL/SLL. CLL/SLL, chronic lymphocytic leukemia/small lymphocytic lymphoma; LN, lymph node; PB, peripheral blood; MOMP, mitochondrial outer membrane permeabilization

The distinct and complementary mechanisms of action make the combination rational. Ibrutinib affects the migration and adhesion, thus blocking the retention and homing of CLL cells [19]. Ibrutinib also inhibits CD40L/IL-21 and CpG mediated proliferation [20]. The most commonly described mechanisms of resistance to ibrutinib are mutations in BTK and PLCG2 [21]. However, non-genetic adaptive mechanisms leading to compensatory pro-survival pathway activation can cause relative resistance to BTK inhibitors. For instance, BCL2 upregulation leads to B cell survival despite BTK inhibition [22]. Venetoclax selectively antagonizes BCL2, induces apoptosis, and sensitizes CLL cells to BTK inhibitors. However, the other two BCL2 family antiapoptotic proteins, MCL1 and BCLX_L_, mediate resistance to venetoclax [23]. Ibrutinib plays a prominent role in downregulating the levels of MCL1 and BCLX_L_ and sensitizes CLL cells to venetoclax [23, 24]. In addition, BTK inhibitors are found to enhance mitochondrial BCL2 dependence in CLL cells without significantly altering overall mitochondrial priming, which results in an increase in pro-apoptotic protein BIM and enhances the killing by venetoclax. On the contrary, venetoclax enhances overall mitochondrial priming without altering BCL2 dependence [25]. The two targeted agents offer complementary functions in the mitochondrial apoptotic pathway. Using an ex vivo model that promotes CLL proliferation, Lu et al. [26] revealed that ibrutinib and venetoclax act on distinct CLL subpopulations that have different proliferative capacities. While ibrutinib preferentially kills the dividing subpopulation, the resting subpopulation responds to venetoclax. It explains the observation that ibrutinib mainly acts on CLL cells in lymph nodes and venetoclax acts on circulating CLL cells. Ibrutinib disrupts adhesion, halts proliferation, and prevents homing of CLL cells in lymph nodes. Without the supporting tumor microenvironment, cells in the periphery become resting and are induced apoptosis by venetoclax. The combination covers both subpopulations of CLL cells, minimizing or even eradicating minimal residual disease (MRD) at all anatomic sites. Kater et al. [27] compared the doublets with single agents in the TCL1 mouse models, resembling aggressive CLL. A combination of decreased proliferation and increased induction of apoptosis are found in the combined treatment, resulting in the deepest responses and longest duration. T cell subsets are also involved in the mechanisms of synergy. The combined therapy results in normalization of the CD4/CD8 ratio and increases naïve cells and reduces effector memory cells in both CD4^+^ and in CD8^+^ cells. The normalization of immune status may help improve the eradication of tumor cells. The mechanisms of synergy in CLL/SLL are shown in Fig. 2.

#### MCL

The combination of ibrutinib and venetoclax displays strong synergistic effects in different MCL cell lines. Ibrutinib rather than venetoclax causes dephosphorylation of BTK(Y223) and AKT(S473), which are associated with survival and proliferation of malignant B-cells. However, enhanced dephosphorylation of the above signal molecules are found in the combined treatment. Similar to CLL, the combination more effectively down-regulates BCL2 family protein compared to single agents. In addition, the combination more efficiently triggers reduced mitochondrial membrane potential and more poly(ADP-ribose) polymerase cleavage [28]. These results illustrate that the synergy is not only due to the distinct mechanisms of action but also mutual promotion between the two agents. Consistent with this conclusion, gene expression profiling demonstrates that not merely transcriptional changes presenting in isolation are enhanced, but emergent transcriptional changes are induced by the combination. Further protein-protein interaction networks reveal activation of apoptosis via p53 and BIM as mechanisms of synergy [29]. Li et al. [30] found that BCL2 expression positively correlates with BTK expression. The high levels of BCL2 result from a defect in protein degradation due to FBXO10 deficiency, as well as transcriptional upregulation through BTK-mediated canonical nuclear factor-κB activation. BTK short hairpin RNA downregulates anti-apoptotic genes including BCL2 and BCLX_L_. The addition of venetoclax could reverse the resistance of MCL cells to ibrutinib in both vitro and vivo. These results reaffirm that BCL2 upregulation is a potential mechanism of resistance to ibrutinib and BCL2 inhibitors can restore the sensitivity of MCL cells to BTK inhibitors.

### Double combination

#### CLL/SLL

The combination of ibrutinib and venetoclax has been used to treat R/R or TN patients with CLL/SLL in some clinical trials and displayed remarkable efficacy. The phase 2 single-arm CLARITY study firstly combined ibrutinib with venetoclax in patients with R/R CLL. In this study, patients initially received 8 weeks of ibrutinib monotherapy. Venetoclax was then added, with a weekly dose ramp-up to the maximum dose of 400 mg/d. The duration of therapy was determined by the peripheral blood (PB) and bone marrow (BM) MRD status at different time points. Overall, 89% of patients responded with 51% achieving CR according to the initial analysis. After 12 months of the combination, 53 and 36% of patients achieved undetected MRD (uMRD) in PB and BM, respectively [31]. And the rate of MRD negativity in BM continued to increase with more cycles of combination therapy. Of 50 evaluable patients, 23 patients stopped both treatments at or before 38 months, mostly due to the achievement of MRD negativity in PB and BM. And the response was sustained after 38 months, although therapy was discontinued in patients with uMRD [32]. The CLARITY study demonstrated that the combination of ibrutinib and venetoclax was highly effective and provided a fixed-duration therapy for R/R patients with CLL. The phase 2 VISION study also investigated the combination of ibrutinib and venetoclax in R/R patients with CLL; however, the study protocol was different from that of the CLARITY study. Patients were initially treated with 2 cycles of ibrutinib monotherapy and venetoclax was added in cycle 3, ramping up to the full dose of 400 mg/d. Patients then received the combination of ibrutinib and venetoclax 400 mg/d for another 12 cycles. After the combination therapy (the end of cycle 15), 36% of patients achieved uMRD in both PB and BM, who were randomized in 2:1 to observation and ibrutinib maintenance. PFS was achieved for 98% of patients randomized to observation at 12 months after randomization. A similar proportion of uMRD patients randomized to ibrutinib maintenance or observation remained uMRD after 12 months (75 and 71%, respectively) [33]. The results suggested that the fixed-duration treatment might provide a durable response for patients with R/R CLL.

In a phase 2 study involving 80 high-risk and older patients with TN CLL, ibrutinib was administered at a standard dose for 3 cycles (28 days each cycle) followed by the addition of venetoclax with a weekly dose escalation to 400 mg once daily. Undetectable MRD in BM was achieved by 56% of patients at 12 cycles and 66% of patients at 24 cycles. The 3-year PFS and OS were 93 and 96%, respectively. Responses were remarkable in patients ≥65 years and across all high-risk subgroups, including unmutated IGHV, TP53 aberration, chromosome 11q deletion, NOTCH1 mutation, and SF3B1 mutation [34]. This study suggested that ibrutinib plus venetoclax could be a very beneficial combination for the frontline treatment of CLL. CPATIVATE is a phase 2 study of first-line ibrutinib combined with venetoclax, which included a MRD cohort and a fixed-duration cohort. Patients received 12 cycles of the combination in both cohorts and those in the MRD cohort were randomized by MRD status to further treatment or placebo. Best uMRD rates were 75% in PB and 68% in BM before randomization [35]. Patients with confirmed uMRD were randomized to placebo or ibrutinib, and 2-year disease-free survival rate post-randomization was 95 and 100%, respectively, with no statistical significance. Patients who did not achieve confirmed uMRD were randomized to ibrutinib or the combination. As a result, greater improvements in best uMRD rates and CR/CRi (CR with incomplete count recovery) rates were observed with the combination than with ibrutinib. Three-year PFS rates were ≥ 95% across all randomized treatment arms [36]. In the fixed-duration cohort, 55% of patients achieved CR/CRi. The 2-year PFS rate was 95% and 2-year OS rate was 98% [37]. The CAPTIVATE study demonstrated that deep and treatment-free remission can be achieved by ibrutinib combined with venetoclax and a fixed-duration regimen. Since the combination is active in both TN and R/R patients with CLL, there seems to be a way out for those who have to take long-term ibrutinib treatment. In a phase 2 study of 45 high-risk patients with CLL receiving at least 1 year of ibrutinib therapy (either as first-line therapy or for R/R disease), venetoclax was added as consolidation and the combination continued for a maximum duration of 2 years. After 12 months of combined therapy, best cumulative rate of uMRD in BM was 73%. Totally, 53% of patients improved their response to CR/CRi during venetoclax treatment. Thirty-five patients completed treatment per protocol and 29 patients achieved uMRD at the completion of planned study treatment [38]. Adding venetoclax to ibrutinib as consolidation may allow discontinuation of indefinite ibrutinib therapy in patients with high-risk CLL.

#### MCL

The phase 2 AIM trial evaluated the combination of ibrutinib and venetoclax in a cohort of 24 patients with MCL, of which 23 were patients with R/R MCL. Ibrutinib was started at a dose of 560 mg once daily for 4 weeks followed by the addition of venetoclax stepwise, weekly increasing doses to 400 mg/d. The CR rate based on computed tomography evaluation at week 16 was 42%, which was significantly higher than the historical result of 9% at this time point with ibrutinib monotherapy. MRD clearance in BM by flow cytometry was achieved in 67% of patients [39]. With a median follow-up of 37.5 months, the median PFS was 29 months. Moreover, some patients in MRD negative 18F-fluorodeoxyglucose-positron-emission tomography (PET)-confirmed CR could discontinue treatment and remain free of progression off therapy [40]. The AIM trial suggested the combination of ibrutinib and venetoclax was highly active in patients with R/R MCL and could be a fixed-duration targeted therapy option for these patients. SYMPATICO is an ongoing phase 3 study evaluating the combination in patients with R/R MCL. The results from the safety run-in period concluded that concurrent ibrutinib and venetoclax were feasible without a lead-in of ibrutinib. With a median follow-up of 31 months, the overall response rate (ORR) was 81%, CR rate was 62%, and the median PFS was 35 months. The response rates were similar regardless of tumor lysis syndrome (TLS) risk. All the patients with positive MRD at baseline achieved uMRD [41]. The ongoing randomized part of this study is investigating the efficacy and safety of ibrutinib plus venetoclax versus ibrutinib plus placebo in patients with R/R MCL. The clinical trials involving the doublets in treating CLL and MCL are summarized in Table 1.

**Table 1:** Clinical trials involving BTK inhibitors combined with BCL2 inhibitors in treating CLL and MCL

Combination	Trial	Phase	Patients	Sample size	Duration of combination	Efficacy	Reference
Ibrutinib+venetoclax	CLARITY EudraCT 2015–003422-14	2	R/R CLL	54	MRD-driven	1-year CR/CRi: 51% 1-year uMRD in PB/BM: 53%/36%	[31]
	VISION NCT03226301	2	R/R CLL	230	1 year	15-month CR: 53% 15-month uMRD in PB/BM: 55%/39%	[33]
	NCT02756897	2	TN CLL/SLL (high-risk and older)	80	2 years	1-year uMRD in BM: 56% 2-year uMRD in BM: 66% 3-year PFS/OS: 93%/96%	[34]
	CAPTIVATE (PCYC-1142) NCT02910583	2	TN CLL/SLL	164	MRD-driven	1-year uMRD in PB/BM: 75%/68% 3-year PFS: ≥95%	[36]
				159	1 year	1-year CR/CRi: 55% 2-year PFS/OS: 95%/98%	[37]
	IMPROVE NCT04754035	2	R/R CLL	38	MRD-driven	2-year uMRD in both PB and BM: 84%	[42]
	NCT03128879	2	CLL/SLL (high-risk and after ibrutinib therapy)	45	MRD-driven	1-year CR/CRi: 53% 1-year uMRD in BM: 73%	[38]
	AIM NCT02471391	2	R/R and TN MCL	24	Until progression or unacceptable toxicity	16-week CR: 42% 16-week uMRD in BM: 67% 1-year PFS/OS: 75%/79%	[39, 40]
	SYMPATICO (PCYC-1143) NCT03112174	3	R/R MCL	21 (safety run-in period)	2 years	31-month CR: 62% 30-month PFS: 60%	[41]
Zanubrutinib+venetoclax	SEQUOIA NCT03336333	3	TN CLL/SLL (high-risk)	80	MRD-driven	Ongoing	/
Acalabrutinib+venetoclax	NCT03946878	2	TN MCL	50	Until progression or unacceptable toxicity	Ongoing	/

*BM* bone marrow; *CLL/SLL* chronic lymphocytic leukemia/small lymphocytic lymphoma, *CR* complete remission, *CRi* CR with incomplete count recovery, *MCL* mantel cell lymphoma, *MRD* minimal residual disease, *OS* overall survival, *PB* peripheral blood, *PFS* progression-free survival, *TN* treatment-naïve, *R/R* relapsed/refractory, *uMRD* undetectable MRD

### Triple combination

The benefit of anti-CD20 monoclonal antibodies added to BTK inhibitors is controversial. Previous studies demonstrated that the addition of rituximab to ibrutinib did not result in higher response rates or longer survival time but indeed shorter time to CR [5, 43]. And this could be attributed to that ibrutinib interferes with rituximab-dependent NK cell-mediated cytotoxicity and antagonizes the anti-tumor activities of rituximab [44]. However, a statistically higher ORR was achieved by ublituximab combined with ibrutinib than ibrutinib monotherapy in high-risk patients with R/R CLL [45]. As compared with rituximab, obinutuzumab utilizes alternative pathways to antibody-dependent cell-mediated cytotoxicity (ADCC), shows an enhanced ADCC effect, and has a higher programmed cell death efficacy [46]. Its superiority has been proved in the CLL11 study in which obinutuzumab was compared head-to-head with rituximab [47]. The addition of obinutuzumab to acalabrutinib seemed to improve both rates and depths of responses as well as PFS in patients with TN CLL [48]. Moreover, it also remains to be determined whether the addition of an anti-CD20 antibody enhances the therapeutic effect of venetoclax. A retrospective comparison showed that the addition of an anti-CD20 antibody was not associated with improvements in ORR, PFS, and OS between the two groups in R/R CLL. Although there was no statistical significance (*P* = 0.07), the incidence of TLS in the combination (5.8%) group was lower than that in the monotherapy group (11.5%) [49]. And another study showed that a pre-induction including 2 cycles of obinutuzumab downgraded the risk of TLS in 25 of 30 patients [50]. Although the benefit of the addition of anti-CD20 monoclonal antibodies to BTK and BCL2 inhibitors remains uncertain, there is an increasing number of clinical trials investigating the triplets in CLL and MCL (Table 2).

**Table 2:** Clinical trials involving BTK inhibitors combined with BCL2 inhibitors and anti-CD20 monoclonal antibodies in treating CLL and MCL

Combination	Trial	Phase	Patients	Sample size	Efficacy	Reference
Ibrutinib+venetoclax+obinutuzumab	NCT02427451	2	TN and R/R CLL	50	uMRD in both PB and BM: 56% (TN) and 44% (R/R) 3-year PFS: 95% in both cohorts 3-year OS: 95% (TN) and 100% (R/R)	[51]
	CLL2-GIVe NCT02758665	2	TN CLL (high-risk)	41	15-month CR: 58.5% 15-month uMRD in PB/BM: 78% /65.9% 2-year PFS/OS: 95.1%/95.1%	[52]
	OAsIs NCT02558816	1/2	TN and R/R MCL	48	6-month CR: 67% (R/R) and 86.6% (TN) 2-year PFS and OS rates (R/R): 69.5 and 68.6% 1-year PFS (TN): 93.3%	[53]
Acalabrutinib+venetoclax+obinutuzumab	NCT03580928	2	TN CLL	44	16-month CR/CRi: 43% 16-month uMRD in PB/BM: 84%/78%	[54]
Acalabrutinib+venetoclax+obinutuzumab or rituximab	CL-003 NCT02296918	1b	TN and R/R CLL/SLL	24	16-month CR/CRi: 50% in both cohorts 10-month uMRD in PB: 67% (R/R) and 75% (TN) 18-month PFS/OS: 100%/100% in both cohorts	[55]
Acalabrutinib+venetoclax+rituximab	NCT02717624	1b	TN MCL	21	6-month CR/PR: 90%/10% 1-year PFS/OS: 89%/95% 6-month uMRD in PB: 75%	[56]
Zanubrutinib+venetoclax+obinutuzumab	NCT03824483	2	TN CLL/SLL	39	26-month uMRD in PB/BM: 95%/89%	[57]
	NCT03824483	2	TN MCL (TP53mut)	25	Ongoing	[58]

*BM* bone marrow, *CLL/SLL* chronic lymphocytic leukemia/small lymphocytic lymphoma, *CR* complete remission, *CRi* CR with incomplete count recovery, *MCL* mantel cell lymphoma, *MRD* minimal residual disease, *OS* overall survival, *PB* peripheral blood, *PFS* progression-free survival, *R/R* relapsed/refractory, *TN* treatment-naïve, *TP53mut* TP53 mutant, *uMRD* undetectable MRD

#### CLL/SLL

In a phase 2 study investigating the triple combination in 25 TN and 25 R/R patients with CLL, obinutuzumab, ibrutinib, and venetoclax were started sequentially and a total of 14 cycles (28 days each cycle) were administered. The ORR was 84% in TN and 88% in R/R patients. Fifty-six percent of TN and 44% of R/R patients achieved uMRD in both PB and BM. The estimated PFS at 36 months is 95% in both groups. The estimated OS at 36 months is 95% for TN and 100% for R/R patients [51]. In the setting of first-line treatment in high-risk CLL with TP53 disruption, the triple combination was given for 6 cycles. Venetoclax was given continuously until cycle 12 and ibrutinib was given until cycle 15 or cycle 36 depending on MRD status. At cycle 15, 58.5% of patients achieved CR, 78% had uMRD in PB, and 65.9% had uMRD in BM. Estimated PFS and OS rates at 24 months were both 95.1% [52]. The second-generation BTK inhibitors, including acalabrutinib and zanubrutinib, do not affect ADCC and are therefore attractive options for combination therapy with anti-CD20 antibodies [59]. Another phase 1b study evaluated the safety and efficacy of acalabrutinib, venetoclax combined with rituximab or obinutuzumab in patients with RR or TN CLL, respectively. At cycle 10, 67% of R/R patients and 75% of TN patients achieved uMRD in PB. Estimated 18-month PFS and OS rates were 100% in both cohorts [55]. Acalabrutinib, venetoclax, and obinutuzumab were highly active in patients with CLL, including high-risk patients. In the phase 2 study by Davids et al. [54], obinutuzumab was administered for 6 cycles and acalabrutinib plus venetoclax were given until cycle 15 or cycle 24 based on MRD status. After 15 cycles of treatment, all of them responded and 78% achieved uMRD in BM. Deep remissions were also seen among patients with TP53 disruption with 70% of them achieving uMRD in BM. MRD-driven treatment with zanubrutinib, obinutuzumab, and venetoclax was investigated in a phase 2 study. Among 37 evaluable patients, 95% achieved uMRD in PB at a median follow-up of 26 months. At a median time of 8 months, 89% of patients achieved uMRD in BM, all of whom met prespecified MRD criteria and discontinued therapy [57].

#### MCL

The triplet combination of ibrutinib, obinutuzumab, and venetoclax was also evaluated in MCL. The phase 1/2 OAsIs study evaluated the maximum tolerated dose of venetoclax in the combination of fixed doses of ibrutinib and obinutuzumab in relapsed MCL patients. The study was then expanded at the maximum tolerated dose of venetoclax in relapsed and untreated MCL patients. A total of 48 patients were enrolled. The CR rate assessed by PET at the end of cycle 6 was 67% in relapsed and 86.6% in untreated patients. MRD clearance in PB was seen in 71.5% of relapsed and 100% of untreated evaluable patients at the end of 3 cycles. And 2-year PFS and OS rates were 69.5 and 68.6% in relapsed patients, respectively. And for untreated patients, 1-year PFS rate was 93.3% [53]. Another phase 1b study reported initial results of acalabrutinib, venetoclax, and rituximab in TN MCL. Rituximab was administered for 6 cycles, followed by maintenance every other cycle for patients achieving CR or PR, through cycle 24. Acalabrutinib was administered continuously and venetoclax was administered for 24 cycles. At the end of cycle 6, ORR was 100% and CR/PR rate was 90%/10% by PET alone. The CR/PR rate by Lugano criteria with BM confirmation was 38%/62%. The 1-year PFS and OS rates were 89 and 95%, respectively. Seventy-five percent of patients with available MRD results at cycle 6 achieved MRD negativity [56]. These studies demonstrated the triplet combination was highly active and may provide durable responses for patients with MCL, especially those untreated patients.

### Side effects

The safety profile of the double combination was similar to what has been noted for ibrutinib or venetoclax monotherapy. For side effects of special interest, atrial fibrillation was reported in 5% ~ 15% of patients [31, 33–35, 39], comparable to that in monotherapy [2, 3, 10, 60]. The number of adult cancer survivors in the United States with comorbid illnesses has increased substantially over the past two decades. Optimal management of comorbid conditions and aggressive interventions for risk reduction are demanded for the cancer survivor population [61]. A population-based study demonstrated that cardiovascular disease mortality risk is highest within the first year after cancer diagnosis, and remains elevated throughout follow-up compared to the general population [62]. Moreover, a pooled analysis revealed that atrial fibrillation incidence was 6.5% for ibrutinib at 16.6-months and 10.4% at the 36-month follow up [63]. Thus, the 2-year-duration combination or MRD-driven pattern is beneficial to avoid the risk of some side effects, such as atrial fibrillation and hypertension. Compared to the double combination, no new side effects were reported in the triple combination. The most common ≥ grade 3 adverse event was neutropenia, occurring in about 30% ~ 50% of the patients [53–55], similar to the data in the trial of ibrutinib combined with venetoclax. Significantly, the application of second-generation BTK inhibitor acalabrutinib depleted cardiotoxicity as atrial fibrillation was reported in one patient in both trials of acalabrutinib, venetoclax, and obinutuzumab [54, 55]. Cooperative effects but nonoverlapping toxicities were seen in the doublets and triplets from the current data and a long-term safety profile is warranted. However, the financial toxicity of the combined treatments should not be ignored. Patients with medical financial hardship have higher risk of cancer mortality and cardiovascular disease mortality [64, 65]. Financial concerns also result in high risk for increased complications among high-risk hematologic malignancy patients. A comprehensive way is effective to intervene on financial toxicity, including navigators, pharmacists, and financial counselors [66].

### Future directions

The combination of BTK and BCL2 inhibitors is highly active, especially in patients with CLL. Although continuous BTK inhibitor use has been demonstrated to be superior to chemoimmunotherapy in TN patients with CLL, it remains to be determined if a fixed-duration treatment with ibrutinib plus venetoclax has a significant advantage over chemoimmunotherapy. The phase 3 GLOW trial has demonstrated that the fixed duration ibrutinib and venetoclax, as compared to chlorambucil plus obinutuzumab, significantly improved response rates, rates of uMRD, and PFS in elderly or unfit patients with TN CLL [67, 68]. A randomized phase 2 trial is currently evaluating ibrutinib plus venetoclax versus fludarabine, cyclophosphamide and rituximab (FCR) in untreated fit patients with CLL. At the end of cycle 9, 71% of patients in the FCR arm and 48% of patients in the combination arm achieved uMRD in BM. The CR/CRi and PR rates were 54 and 46% in the FCR arm and 76 and 24% in the combination arm, respectively. However, the uMRD rate may improve after longer exposure to the combination and the results at cycle 27 will be decisive in determining the best therapeutic strategy [69]. It also remains unknown whether the combination of BTK and BCL2 inhibitors is superior to BTK inhibitor monotherapy or BCL2 inhibitor plus a CD20-antibody in patients with CLL. And the advantage of the triplet combinations over the doublet combinations has to be demonstrated in the future. Several ongoing randomized trials are currently evaluating the BTK and BCL2 inhibitor combination therapies (Table 3). The results of these studies may address these questions in the future.

**Table 3:** Comparative studies involving chemoimmunotherapy and combinations of novel agents

Comparisons	Trial	Phase	Patients
FCR vs I + V	NCT04010968	2	TN CLL(fit)
FCR/BR vs V + R vs V + O vs I + V + O	NCT02950051	3	TN CLL(fit)
FCR/BR vs A + V ± O	NCT03836261	3	TN CLL
I + V vs Chl + O	NCT03462719	3	TN CLL/SLL
I + O vs I + O + V	NCT03737981	3	TN CLL/SLL(older)
	NCT03701282	3	TN CLL/SLL(younger)
I vs I + V vs V + O	NCT04608318	3	TN CLL
A + V vs V + O	NCT05057494	3	TN CLL/SLL

*A* acalabrutinib, *BR* bendamustine and rituximab, *Chl* chlorambucil, *CLL/SLL* chronic lymphocytic leukemia/small lymphocytic lymphoma, *FCR* fludarabine, cyclophosphamide and rituximab, *I* ibrutinib, *O* obinutuzumab, *R* rituximab, *V* venetoclax, *TN* treatment-naïve

Although combinations of BTK and BCL2 inhibitors are associated with durable responses in patients with CLL, relapses could be inevitable in a proportion of patients. Early MRD kinetics were used to predict delayed uMRD and earlier post-treatment MRD recurrence. In the study of zanubrutinib, obinutuzumab, and venetoclax, patients who failed to have a ≥ 400-fold reduction in PB MRD after 4 cycles (ΔMRD400) experienced delayed BM MRD clearance and earlier MRD recurrence, despite longer treatment duration. ΔMRD400 warrants further study as a predictive biomarker for treatment duration [57]. Since agents with the most active mechanisms have been used first, what will we use to treat patients who develop disease relapse. For those who have stopped therapies, retreatment with combinations of BTK and BCL2 inhibitors would probably lead to a second response. Data from the MURANO study suggested that 55% of relapsed patients would respond to venetoclax retreatment [70]. A longer follow-up will be needed to determine if retreatment with these agents would result in a second durable response in the relapsed patients. For those who received BTK inhibitor maintenance due to MRD positivity, a BCL2 inhibitor plus an anti-CD20 monoclonal antibody or alternative treatment could be used in the relapsed setting. Various mechanisms underlying the resistance to ibrutinib or venetoclax monotherapy have been described in detail [71]. Elaboration of mechanisms underlying the resistance to the combinations would provide insights for the development of novel treatments for patients who relapse after the combination therapy.

## Conclusions

The combinations of BTK inhibitors and venetoclax with or without anti-CD20 monoclonal antibodies are highly active and well-tolerated and provide fixed-duration options for patients with CLL and MCL. Data from the ongoing randomized trials may confirm the superiority of these regimens in the future.

## Data Availability

Not applicable.

## Abbreviations

CLL/SLL: Chronic lymphocytic leukemia/small lymphocytic lymphoma
MCL: Mantle cell lymphoma
R/R: Relapsed/refractory
CR: Complete remission
PFS: Progression-free survival
OS: Overall survival
TN: Treatment-naïve
PR: Partial remission
MRD: Minimal residual disease
PB: Peripheral blood
BM: Bone marrow
uMRD: Undetected MRD
CRi: CR with incomplete count recovery
PET: 18F-fluorodeoxyglucose-positron-emission tomography
ORR: Overall response rate
TLS: Tumor lysis syndrome
ADCC: Antibody-dependent cell-mediated cytotoxicity
FCR: Fludarabine, cyclophosphamide and rituximab
